# Impact of structural and interpersonal racism on oral health and dental services

**DOI:** 10.11606/s1518-8787.2026060007164

**Published:** 2026-03-16

**Authors:** Rafaela de Oliveira Cunha, Isabel Cristina Gonçalves Leite, Mário Círio Nogueira

**Affiliations:** I Universidade Federal de Juiz de Fora. Faculdade de Medicina. Programa de Pós-Graduação em Saúde Coletiva. Juiz de Fora, MG, Brasil; II Universidade Federal de Juiz de Fora. Faculdade de Medicina. Departamento de Saúde Coletiva. Juiz de Fora, MG, Brasil

**Keywords:** Racism, Residential Segregation, Residence Characteristics, Oral Health, Health Services Accessibility

## Abstract

**OBJECTIVE:**

To analyze the role of racism in oral health and access to dental services, considering two impact pathways: structural racism and interpersonal racism.

**METHODS:**

A cross-sectional study with 686 undergraduate students, which analyzed two categories of outcomes: those related to oral health (self-perception, oral health impairment, and tooth loss) and those related to access to/perception of the quality of dental services (access to services, recent use, and evaluation of care). Exposures were race/color, racial discrimination, and characterization of the neighborhood environment. Prevalence ratios were estimated using Poisson regression with robust variance, with adjustments guided by directed acyclic diagrams.

**RESULTS:**

Black and brown people had a higher prevalence of negative self-perception (black: PR = 1.56; 95%CI 1.14–2.13; brown: PR = 1.53; 95%CI 1.14–2.05), tooth loss (black: PR = 1.99; 95%CI 1.33–2.97), lack of access to dental services (black: PR = 3.08; 95%CI 1.06–8.95; brown: PR = 3.78; 95%CI 1.49–9.59), and negative evaluation of care (black: PR = 1.63; 95%CI 1.08–2.45; brown: PR = 1.64; 95%CI 1.12–2.39). Racial discrimination was associated with lack of access (PR = 4.34; 95%CI 1.45–12.95), recent non-use of services (PR = 1.74; 95%CI 1.31–2.31) and negative evaluation of care (PR = 1.51; 95%CI 1.01–2.25). And living in neighborhoods with fewer physical and social problems was associated with a lower prevalence of negative self-perception (PR = 0.70; 95%CI 0.54–0.90), compromised oral health (PR = 0.87; 95%CI 0.76–0.99), lack of access (PR = 0.42; 95%CI 0.18–0.98), and negative evaluation of care (PR = 0.56; 95%CI 0.40–0.79).

**CONCLUSIONS:**

The findings show that racism impacts oral health through structural and interpersonal channels and highlight the urgency of broad and effective strategies to reduce the inequities affecting racialized groups.

## INTRODUCTION

Racial inequities in oral health are widely documented, with evidence indicating that individuals from racialized groups face greater barriers to access and quality of dental care, as well as having a higher prevalence of conditions such as caries, periodontal disease, and tooth loss^1,2^. The literature has highlighted the importance of understanding race as a social construct and investigating racism as a fundamental cause of these inequities^3,4^.

Racism, as a complex social system, operates on multiple levels. At the structural level, it is expressed in the historical and institutional organization of society, through policies, norms, practices, and institutional arrangements that produce and reproduce racial hierarchies, conditioning unequal access to resources, opportunities, and rights. At the interpersonal level, it manifests itself in discriminatory attitudes and behaviors that directly affect racialized individuals, perpetuating experiences of exclusion and subordination^5^. This system impacts health through a number of ways, including: (I) economic injustice and social segregation, which generate precarious living conditions; (II) unequal exposure to adverse physical and social environments; and (III) psychosocial trauma resulting from interpersonal discrimination, with effects on mental, behavioral, and physical health^6^. Thus, place of residence and life experiences are closely linked to the state of health of individuals^3^.

Despite the consistent evidence on racial inequities in oral health, the role of racism in producing these inequities remains underexplored. Most studies adopt descriptive approaches based solely on self-identified race, which restricts understanding of the mechanisms by which racism influences oral health outcomes^3^. Research that goes beyond this perspective and examines the ways in which racism influences oral health and access to services is essential to guide public policies aimed at equity.

In this context, this study aimed to investigate the impact of racism on oral health and access to dental services, considering two dimensions of this system: interpersonal racism, measured by the experience of self-reported racial discrimination, and structural racism, represented by the characteristics of the neighborhood environment, which express residential segregation, socio-spatial inequality, and the historical concentration of disadvantages. Understanding these relationships helps to identify the mechanisms that produce racial inequalities in oral health and provide subsidies for more equitable public policies and intervention strategies.

## METHODS

### Design, Study Population, and Ethical Aspects

A cross-sectional study was carried out with undergraduate students enrolled in 2023 at the Universidade Federal de Juiz de Fora (UFJF), a public institution based in Juiz de Fora (MG) and an advanced campus in Governador Valadares (MG). All students aged 18 or over were eligible, and those who did not respond to the survey questionnaire after three attempts to contact them were considered to have lost the sample.

The choice of the university population is based on the racial, socioeconomic, and territorial diversity present in the academic environment, which favors the analysis of racial inequities in a plural institutional context. Although still marked by inequalities, the university space has become more heterogeneous with the implementation of affirmative actions. This scenario makes it possible to investigate the persistence of racial inequalities even in contexts of higher education, as well as highlighting the effects of racism on trajectories marked by different social exposures and opportunities.

In 2023, UFJF had 15,361 students enrolled in face-to-face undergraduate courses, 87.6% of them on the Juiz de Fora campus. Of these, 59.4% declared themselves white, 27.5% brown, 10.7% black, and 2.4% yellow, indigenous, or of another race/color. The sample size was calculated for finite populations, estimating a minimum of 347 participants, based on an expected prevalence of racial discrimination of 9%^7^, a 95% confidence interval and a sampling error of 5%.

The study was approved by the UFJF Human Research Ethics Committee (opinion no. 6.041.263).

### Data Collection

Data was collected using an online questionnaire, prepared on the Google Forms platform and sent to the students by institutional e-mail. The instrument contained 54 questions, organized into four blocks: Block I - Demographic and socioeconomic data; Block II - Oral health; Block III - Neighborhood Perception Scale^8^; Block IV - Abbreviated Explicit Discrimination Scale (EDS)^9^.

The Neighborhood Perception Scale, validated in Brazil, evaluates the perception of the environment considering physical and social disorders, with a score from 0 to 32, with higher values indicating greater problems in the neighborhood^8^. The abbreviated EDS is a validated instrument that measures the experience of explicit discrimination in the Brazilian context. Its abbreviated version, considered suitable for undergraduate students^9^, measures experiences of discrimination in eight everyday situations, with scores ranging from 0 to 24, with higher values indicating greater perceived discrimination.

### Variables

This study adopted an analytical strategy aimed at articulating different dimensions of racism. The characterization of the neighborhood environment was used as a proxy for structural racism, considering that territorial inequalities express the historical and institutional materialization of racial hierarchies in the urban space^6^. Self-reported discrimination was considered a measure of interpersonal racism, reflecting the direct experiences of differentiated treatment and prejudice experienced by individuals in everyday interactions^9^. This operationalization made it possible to simultaneously capture different levels of racism (structural and interpersonal) and analyze its effects on oral health outcomes and access to dental services.

Thus, the independent variables included: race/color, racial discrimination, and neighborhood characterization.

The race/color variable was obtained according to the participants’ self-declaration, based on the Instituto Brasileiro de Geografia e Estatística (IBGE — Brazilian Institute of Geography and Statistics) classification: white, black, brown, yellow, and indigenous. Racial discrimination was measured using the abbreviated version of the EDS, dichotomized as “yes” or “no”. Individuals who reported at least one episode of unequal treatment based on race/color and interpreted it as discriminatory were considered discriminated against. Neighborhood characterization was measured using the Neighborhood Perception Scale and dichotomized into “fewer physical and social problems” (lower tertile) and “more physical and social problems” (middle and upper tertiles)^10^. Considering that many students migrate from one city to another to attend university, the neighborhood where the participant had lived for most of their life or for at least five years was considered, to capture long-term experiences that influenced their life opportunities.

Two categories of outcomes were analyzed: outcomes related to oral health (self-perceived oral health, oral health impairment, and tooth loss) and outcomes related to access to and perceived quality of dental services (access to dental services, recent use of dental services, and evaluation of care).

Self-perception of oral health was classified as positive or negative, according to the respondent’s own assessment. Oral health impairment was identified by the presence of at least one of the following conditions: toothache, discomfort with oral appearance, or eating difficulties, with the answers “very often”, “often”, or “sometimes” being considered indicative of impairment^11^. Tooth loss was defined by the report of extraction of at least one permanent tooth, categorized as “yes” or “no”.

The variables related to access to and perception of the quality of dental services were based on the Projeto SB Brasil 2020 (2020 OH Brazil Project) questionnaire. Access was classified as “access” for the answers “sought and was scheduled” or “was attended to”; and “lack of access” for the answer “was not attended to”. Recent use of dental services was defined as having seen a dentist in the last year, while recent non-use included cases where the last visit was more than a year ago or when the individual had never been to the dentist. The evaluation of dental care was categorized as “positive” or “negative”.

The covariates included age, gender, marital status, family income, maternal schooling, paid work, type of high school, receipt of student aid, and subject area.

### Statistical Analysis

The analyses were carried out using R software (version 4.2.1). Initially, a descriptive analysis was carried out. Poisson regression with robust variance was used to estimate prevalence ratios. This approach is recommended in cross-sectional studies with binary outcomes, as it provides more accurate estimates of prevalence ratios^12^. Confidence intervals of 95% (95%CI) were adopted.

The variables included in the adjusted models were selected based on directed acyclic diagrams (DAGs), built in the DAGitty program, according to theoretical assumptions and empirical evidence from the literature^4^. The DAGs were built from an adaptation of the model proposed by Howe et al.^4^, which originally addressed different health outcomes (health behaviors, body mass index – BMI, and physical conditions). For this study, conceptual and analytical adaptations were made for the context of oral health and access to dental services, allowing specific causal relationships to be represented for these outcomes. Latent variables were incorporated to capture unobservable factors with a possible causal influence. Contextual variables relating to the provision of services (which can influence access and oral health) were not included in the diagrams due to the unavailability of these data in the study population.

The fits of the Poisson regression models were assessed for the presence of overdispersion (when the variance is greater than the mean, identified by the dispersion statistic > 1.2), the presence of multicollinearity (by the VIF statistic > 5, “variance inflation factor”) and the distribution of Pearson and Deviance residuals without major deviations (within the range of -0.5 +0.5).

The Figure shows the DAG with the study’s exposures, outcomes and covariates.

**Figure:**
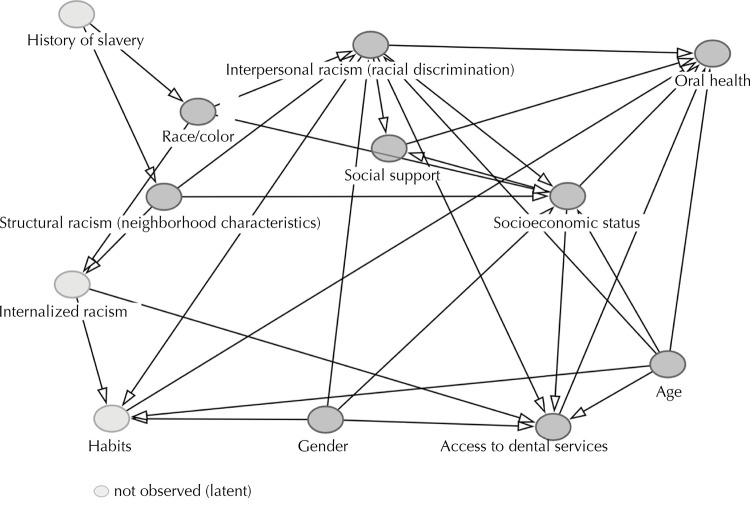
Causal diagram of the relationships between exposures, outcomes and study covariates.

## RESULTS

The study included 686 undergraduate students. The yellow and indigenous categories were excluded from the analysis due to their low representativeness (one indigenous and no yellow). The age distribution was 17.9% between 18 and 20 years old, 51.9% between 21 and 24 years old, and 30.2% aged 25 or over. The majority were female (67.9%) and had a monthly family income of up to three minimum wages (47.7%). As for race/color, 58.2% declared themselves white, 23.2% brown and 18.6% black.

Table 1 shows the characteristics of the study population, stratified by race/color. There was a higher proportion of black and brown individuals in the lower family income categories compared to white (p < 0.001). The frequency of enrollment in public high schools was also significantly higher among black and brown people (p < 0.001). Black and brown individuals reported a higher frequency of living in neighborhoods with more physical and social problems, compared to white (p < 0.001). And the prevalence of reported racial discrimination was also higher among black individuals (p < 0.001).

**Table 1:** Characteristics of the study population: total distribution and by race/color, in absolute values and percentages (n = 686).

Variable	Total		Race/color						p value
			White		Brown		Black		
	n	%	n	%	n	%	n	%	
Gender									0.283
Cisgender female	466	67.9	261	56.0	116	24.9	89	19.1	
Cisgender male	189	27.6	118	62.4	35	18.5	36	19.0	
Transgender, agender, or non-binary	31	4.5	20	64.5	8	25.8	3	9.7	
Age									0.097
Less than 20 years old	123	17.9	72	58.5	32	26.0	19	15.4	
Between 20 and 24 years old	356	51.9	221	62.1	71	19.9	64	18.0	
Over 25 years old	207	30.2	106	51.2	56	27.1	45	21.7	
Marital status									0.073
With spouse	76	11.1	36	47.4	25	32.9	15	19.7	
No spouse	610	88.9	363	59.5	134	22.0	113	18.5	
Monthly family income									< 0.001
From 0 to 3 minimum wages	327	47.7	142	43.4	88	26.9	97	29.7	
Between 3 and 5 minimum wages	158	23.0	108	68.4	35	22.2	15	9.5	
5 minimum wages or more	201	29.3	149	74.1	36	17.9	16	8.0	
Maternal schooling									< 0.001
Up to complete elementary school	169	24.6	75	44.4	55	32.5	39	23.1	
Incomplete or complete high school	223	32.5	120	53.8	49	22.0	54	24.2	
Incomplete higher education or more	294	42.9	204	69.4	55	18.7	35	11.9	
Work									0.246
No	501	73.0	301	60.1	111	22.2	89	17.8	
Yes	185	27.0	98	53.0	48	25.9	39	21.1	
Type of secondary school									< 0.001
Private	282	41.1	215	76.2	40	14.2	27	9.6	
Public	404	58.9	184	45.5	119	29.5	101	25.0	
Admission to UFJF by racial quotas									< 0.001
No	532	77.6	399	75.0	89	16.7	44	8.3	
Yes	154	22.4	0	0	70	45.5	84	54.5	
Student aid									< 0.001
No	554	80.8	343	61.9	126	22.7	85	15.3	
Yes	132	19.2	56	42.4	33	25.0	43	32.6	
Area of knowledge of the course									0.556
Health and Biological Sciences	241	35.1	141	58.5	55	22.8	45	18.7	
Exact Sciences and Engineering	91	13.3	60	65.9	17	18.7	14	15.4	
Humanities and Social Sciences	354	51.6	198	55.9	87	24.6	69	19.5	
Experience of racial discrimination									
No	436	63.6	356	81.7	72	16.5	8	1.8	< 0.001
Yes	250	36.4	43	17.2	87	34.8	120	48.0	
Neighborhood characterization									< 0.001
More problems	309	45.0	153	49.5	85	27.5	71	23.0	
Fewer problems	377	55.0	246	65.3	74	19.6	57	15.1	

UFJF: Universidade Federal de Juiz de Fora.

Among the students who reported interpersonal discrimination, 92.3% attributed the unequal treatment to multiple reasons, the most cited being the way they dressed, socioeconomic status and race/color. Specifically regarding racial discrimination, 36.4% reported this experience. The most frequent occurrences included: inferior treatment in commercial establishments (21.0%), being mistaken for an employee (17.1%) and verbal insults (18.1%). Discrimination was also reported in educational institutions (12.1%), in emotional relationships (10.9%), in the neighborhood (7.4%), in public offices (6.7%), and in the family (6.9%).

Table 2 shows the oral health outcomes and access to dental services. Oral health was compromised in 57.3% of the participants; 25.7% reported negative self-perception and 16.5% tooth loss. As for access, 3.9% reported a lack of access; 69.0% had used dental services in the last year, and 17.3% gave a negative assessment of the care they received.

**Table 2:** Prevalence of oral health outcomes and access to dental services: total distribution and by race/color (n = 686).

Outcome	Total		Race/Color					
			White		Brown		Black	
	n	% (95%CI)	n	% (95%CI)	n	% (95%CI)	n	% (95%CI)
Oral health								
Negative self-perception of oral health	176	25.7 (22.4–29.0)	81	20.3 (16.4–24.2)	52	32.7 (25.4–40.0)	43	33.6 (25.4–41.8)
Compromised oral health	393	57.3 (53.6–61.0)	225	56.4 (51.5–61.3)	92	57.9 (50.3–65.5)	76	59.4 (51.0–67.8)
Tooth loss	113	16.5 (13.8–19.2)	50	12.5 (9.2–15.8)	30	18.9 (12.8–25.0)	33	25.8 (18.2–33.4)
Access to dental services								
Lack of access to dental services	27	3.9 (2.5–5.3)	7	1.8 (0.4–3.2)	12	7.5 (3.4–11.6)	8	6.3 (2.2–10.4)
No recent use of dental services	213	31.0 (27.5–34.5)	114	28.6 (24.1–33.1)	52	32.7 (25.4–40.0)	47	36.7 (28.3–45.1)
Negative evaluation of dental care	119	17.3 (14.6–20.0)	52	13.0 (9.7–16.3)	37	23.3 (16.6–30.0)	30	23.4 (16.1–30.7)

95%CI: 95% confidence interval.

Table 3 shows the prevalence ratios (PR) between exposure variables and oral health-related outcomes. After adjusting for confounders indicated by the DAG, race/color remained associated (p < 0.05) with negative self-perception of oral health (PR = 1.56; 95%CI 1.14–2.13 for black and PR = 1.53; 95%CI 1.14–2.05 for brown individuals) and tooth loss (PR = 1.99; 95%CI 1.33–2.97 for black individuals). Experiencing racial discrimination was associated with worse oral health outcomes only in the crude analysis, with an association with negative self-perception of oral health (PR = 1.49; 95%CI 1.16–1.91) and tooth loss (PR = 1.78; 95%CI 1.27–2.48). Finally, living in areas with fewer physical and social problems was a protective factor for negative self-perception (PR = 0.70; 95%CI 0.54–0.90) and oral health impairment (PR = 0.87; 95%CI 0.76–0.99), even after adjusting for confounding factors (p < 0.05).

**Table 3:** Prevalence ratios of oral health-related outcomes by race/color, experience of racial discrimination and neighborhood characterization (n = 686).

Variable	Negative self-perception of oral health		Compromised oral health		Tooth loss	
	Crude PR (95%CI)	Adjusted PR (95%CI)	Crude PR (95%CI)	Adjusted PR (95%CI)	Crude PR (95%CI)	Adjusted PR (95%CI)
Race/color						
White	1	1	1	1	1	1
Brown	1.61 (1.20–2.17)	1.53 (1.14–2.05)	1.03 (0.88–1.20)	1 (0.86–1.18)	1.51 (1.00–2.28)	1.46 (0.96–2.23)
Black	1.65 (1.21–2.26)	1.56 (1.14–2.13)	1.05 (0.89–1.24)	1.03 (0.87–1.22)	2.06 (1.39–3.04)	1.99 (1.33–2.97)
Racial discrimination^b^						
No	1	1	1	1	1	1
Yes	1.49 (1.16–1.91)	1.07 (0.77–1.47)	1.08 (0.94–1.23)	1.05 (0.87–1.25)	1.78 (1.27–2.48)	1.18 (0.78–1.79)
Neighborhood characterization^c^						
More problems	1	1	1	1	1	1
Fewer problems	0.65 (0.50–0.84)	0.70 (0.54–0.90)	0.87 (0.76–0.99)	0.87 (0.76–0.99)	0.75 (0.54–1.05)	0.82 (0.58–1.16)

PR: prevalence ratio; 95%CI: 95% confidence interval; DAG: directed acyclic diagrams.

^a^Prevalence ratio adjusted for race by neighborhood characterization, according to DAG.

^b^Prevalence ratio adjusted for racial discrimination by gender, age, neighborhood characterization, and race/color, according to DAG.

^c^Prevalence ratio adjusted for neighborhood characterization by race/color, according to DAG.

Note: the Poisson regression model adjustments were assessed for the presence of overdispersion (dispersion statistic > 1.2), multicollinearity (VIF > 5) and the distribution of Pearson and Deviance residuals, which remained close to zero, indicating a good model fit.

Table 4 shows the associations with the outcomes of access and perceived quality of dental services. In the adjusted analysis, race/color remained associated (p < 0.05) with lack of access and negative evaluation of care. Black (PR = 3.08; 95%CI 1.06–8.95) and brown (PR = 3.78; 95%CI 1.49–9.59) individuals had a higher prevalence of lack of access and negative evaluation of the care received (black: PR = 1.63; 95%CI 1.08–2.45; brown: PR = 1.64; 95%CI 1.12–2.39). The experience of racial discrimination was associated with lack of access (PR = 4.34; 95%CI 1.45–12.95), lack of recent use of services (PR = 1.74; 95%CI 1.31–2.31), and negative evaluation of care (PR = 1.51; 95%CI 1.01–2.25) (p < 0.05). And living in neighborhoods with fewer problems was a protective factor for both lack of access (PR = 0.42; 95%CI 0.18–0.98) and negative evaluation of care (PR = 0.56; 95%CI 0.40–0.79) (p < 0.05). It should be noted that some of the associations observed, especially between lack of access to dental services, race/color, and racial discrimination, although statistically significant, had wide confidence intervals, indicating less precision in the estimates. This imprecision is mainly due to the low prevalence of the outcome in the population studied, which reduces the number of events and increases the uncertainty of the effect measures. Even so, the consistency of the direction of the associations reinforces the presence of racial inequalities in access to services, even in the face of sampling and statistical limitations.

**Table 4:** Prevalence ratios of outcomes related to access to dental services by race/color, experience of racial discrimination and neighborhood characterization (n = 686).

Variable	Lack of access to dental services		Lack of recent use of dental services		Negative evaluation of dental care	
	Crude PR (95% CI)	Adjusted PR (95%CI)	Crude PR (95%CI)	Adjusted PR (95%CI)	Crude PR (95%CI)	Adjusted PR (95%CI)
Race/color						
White	1	1	1	1	1	1
Brown	4.30 (1.72–10.7)	3.78 (1.49–9.59)	1.14 (0.87–1.50)	1.14 (0.87–1.50)	1.79 (1.22–2.61)	1.64 (1.12–2.39)
Black	3.56 (1.32–9.63)	3.08 (1.06–8.95)	1.29 (0.98–1.69)	1.28 (0.97–1.69)	1.80 (1.20–2.69)	1.63 (1.08–2.45)
Racial discrimination^b^						
No	1	1	1	1	1	1
Yes	6.1 (2.50–14.92)	4.34 (1.45–12.95)	1.54 (1.24–1.92)	1.74 (1.31–2.31)	1.96 (1.42–2.71)	1.51 (1.01–2.25)
Neighborhood characterization^c^						
More problems	1	1	1	1	1	1
Fewer problems	0.35 (0.15–0.78)	0.42 (0.18–0.98)	0.96 (0.77–1.2)	0.99 (0.79–1.24)	0.52 (0.37–0.72)	0.56 (0.40–0.79)

PR: prevalence ratio; 95%CI: 95% confidence interval; DAG: directed acyclic diagrams.

^a^Prevalence ratio adjusted for race by neighborhood characterization, according to DAG.

^b^Prevalence ratio adjusted for racial discrimination by gender, age, neighborhood characterization, and race/color, according to DAG.

^c^Prevalence ratio adjusted for neighborhood characterization by race/color, according to DAG.

Note: the fits of the Poisson regression models were assessed for the presence of overdispersion (dispersion statistic > 1.2), multicollinearity (VIF > 5) and the distribution of Pearson and Deviance residuals, which remained close to zero, indicating a good fit of the model.

## DISCUSSION

The findings of this study confirm significant racial inequalities in oral health and access to dental services, with racism operating interpersonally and structurally in its production. It was observed that individuals who declared themselves black had a higher prevalence of negative self-perception of oral health, tooth loss, lack of access to dental services, and negative evaluation of care, compared to whites. Among brown individuals, there was a higher prevalence of negative self-perception of oral health, lack of access to dental services, and negative evaluation of care, also compared to whites.

Being a part of historically marginalized racial groups exposes individuals to higher levels of interpersonal discrimination and adverse social contexts, both of which have an impact on health^4^. In this study, racial discrimination was associated with oral outcomes only in the crude analysis, losing significance after adjustment, a result that is in line with the heterogeneity observed in the literature. While studies such as those by Celeste et al.^13^ and Finlayson et al.^14^ found no significant associations, other studies, such as those by McGlumphy et al.^15^, Cozier et al.^16^ and Raskin et al.^17^, identified a positive relationship between experiences of racial discrimination and worse oral health outcomes, such as negative self-perception and tooth loss. These effects can be mediated by psychological suffering and the adoption of unhealthy behaviors, such as smoking, alcohol abuse, and excessive sugar intake, as well as restrictions on access to educational, employment and housing opportunities, which increase exposure to environmental risks and limit the use of dental services^18^.

This study also contributes to understanding the effects of racial discrimination on access to dental services. There was an association between experiences of discrimination, less use of services, and negative evaluations of care, in line with previous studies^17,19^. Discrimination can affect users’ perceptions of the health system, impair interactions with professionals and compromise both access to and quality of care. Discriminatory situations in clinical settings tend to generate feelings of alienation, reinforce paternalistic or coercive practices, and impose cultural and communication barriers, which fuels distrust in the system, reduces adherence to treatment, and contributes to dissatisfaction and the persistence of unmet needs^19^.

It is important to note that such attitudes do not occur in isolation but reflect social structures that sustain racism. Beyond individual behaviors, racism is rooted in the political and economic organization of society, operating as a social norm reproduced by historically established power structures^3^. The literature recognizes structural racism as the central determinant of racial inequities in health^6^. In this sense, understanding these inequities requires attention to the historical and contextual conditions that sustain them, among which residential segregation stands out as a fundamental mechanism of structural racism^20^.

In Brazil, although residential segregation is not instituted by formal racial segregation policies, it is widely manifested through the exclusion of black populations from the central and more developed areas of cities^20^. Thus, racialized populations, often in situations of socioeconomic vulnerability, tend to live in areas marked by a lack of resources, such as healthy food, quality education, and employment opportunities, and face greater obstacles in accessing adequate health services^21^.

Thus, the neighborhood environment is a relevant marker for understanding the effects of structural racism on health. Individuals living in the same neighborhood share physical, social, and institutional conditions that affect their health, in addition to their individual characteristics^22^. In the study of structural racism, the spatial concentration of health behaviors and outcomes in hyperlocal areas reflects the context of the neighborhood, which is strongly influenced by racial segregation and the institutional practices that have historically organized urban space along racial lines^23^. Mohottige et al.^24^ showed that indicators of structural racism at the neighborhood level, such as racial segregation and socioeconomic deprivation, were associated with a higher prevalence of chronic diseases, including diabetes, hypertension, and chronic kidney disease, reinforcing the role of the neighborhood as a concrete expression of structural racism.

The findings of this study confirm that living in areas with better physical and social conditions acts as a protective factor for negative oral health outcomes, such as negative self-perception and oral health impairment, as well as for barriers to accessing dental services, including lack of access and negative evaluation of care, even after adjusting for race/color. The literature argues that living in disadvantaged areas is often associated with an imbalance between protective and risk factors for health. These areas tend to have less available dental services, poor infrastructure, and an overload of risk factors for oral diseases, creating a scenario of vulnerability that compromises the quality of life of their residents^25,26^. The shortage of professionals and care units, coupled with financial, institutional, and cultural barriers, hinders access to quality preventive and curative care. Furthermore, the lack of effective public policies aimed at the equitable distribution of health services exacerbates these inequities, reinforcing racial and socioeconomic inequities in access to dental care^25,27^. These impacts are intensified by exposure to environmental and behavioral risk factors, such as diets rich in sugars, limited health education, and the prevalence of harmful habits such as smoking and excessive alcohol consumption, which also contribute to an increased risk of oral diseases^26^.

Therefore, the neighborhood must be understood not only as a living context, but as a concrete expression of historically reproduced structural inequalities that shape opportunities, resources and health conditions^22^. By reflecting racial segregation and the unequal organization of urban space, the neighborhood environment acts as a fundamental mediator of inequities in oral health and access to dental services between different racial groups.

Furthermore, it must also be recognized that racism does not act in isolation but intertwines with other forms of oppression to shape oral health and access to dental services^3^. The intersectional perspective allows us to understand these complex interactions, highlighting how multiple axes of oppression combine to impact historically marginalized groups^11^. Studies emphasize that racism, sexism, classism, and other systems of marginalization are interconnected, co-constituting each other and being central to racial and non-racial inequities in health^11,28^. Thus, individuals marginalized in multiple axes of oppression often have less power, less control over their environments, reduced ability to avoid oppressive contexts and fewer resources to deal with oppression, factors that together may explain the negative health effects observed in some groups^11^. This understanding reinforces the urgency of public policies and interventions that understand and tackle the multiple forms of oppression, promoting oral health equity.

Some limitations of this study should be considered. As it is based on self-reported outcomes, there is a risk of response and memory bias, which could compromise the accuracy of the information reported. The use of a non-probabilistic sample limits the generalization of the findings to the university population as a whole. And the cross-sectional design prevents the inference of direct causal relationships. Despite this, the study makes relevant contributions by highlighting the effects of interpersonal and structural racism on oral health and access to dental services. By considering two important impact pathways of racism, the study broadens the understanding of the determinants that sustain racial inequities in this field.

The findings show that structural and interpersonal racism hinder access to quality dental care for marginalized racial groups and contribute to worsening oral health conditions. Overcoming these inequities requires a comprehensive approach aimed at transforming the social and economic conditions that perpetuate them. This includes formulating and implementing public policies that combat discriminatory practices, tackle spatial segregation, promote healthy neighborhood environments, and guarantee an equitable supply of quality dental services, with special attention to historically excluded populations. Only through structural changes will it be possible to mitigate racial inequalities in oral health and move towards a fairer and more equitable society.
