# Supplementary material for: Impact of structural and interpersonal racism on oral health and dental services
Source: Rev Saude Publica. 2026 Mar 16;60:e11. doi: 10.11606/s1518-8787.2026060007164 (PMC12991402; doi:10.11606/s1518-8787.2026060007164)
Supplement: Translation

# Translation: Impacto do racismo estrutural e interpessoal na saúde bucal e nos serviços odontológicos

**DOI:** 10.11606/s1518-8787.2026060007164

**Authors:** Rafaela de Oliveira Cunha, Isabel Cristina Gonçalves Leite, Mário Círio Nogueira

**Affiliations:** I Universidade Federal de Juiz de Fora. Faculdade de Medicina. Programa de Pós-Graduação em Saúde Coletiva. Juiz de Fora, MG, Brasil; II Universidade Federal de Juiz de Fora. Faculdade de Medicina. Departamento de Saúde Coletiva. Juiz de Fora, MG, Brasil

**Keywords:** Racismo, Segregação Residencial, Características de Residência, Saúde Bucal, Acesso aos serviços de saúde

## Abstract

**OBJETIVO:**

Analisar o papel do racismo na saúde bucal e no acesso a serviços odontológicos, considerando duas vias de impacto: o racismo estrutural e o racismo interpessoal.

**MÉTODOS:**

Estudo transversal com 686 estudantes de graduação, que analisou duas categorias de desfechos: os relacionados à saúde bucal (autopercepção, comprometimento da saúde bucal e perda dentária) e os relacionados ao acesso/à percepção da qualidade de serviços odontológicos (acesso aos serviços, uso recente e avaliação do atendimento). As exposições foram raça/cor, discriminação racial e caracterização do ambiente de vizinhança. Foram estimadas as razões de prevalência através de regressão de Poisson com variância robusta, com ajustes orientados por diagramas acíclicos direcionados.

**RESULTADOS:**

Pretos e pardos apresentaram maior prevalência de autopercepção negativa (pretos: RP = 1,56; IC95% 1,14–2,13; pardos: RP = 1,53; IC95% 1,14–2,05), perda dentária (pretos: RP = 1,99; IC95% 1,33–2,97), falta de acesso aos serviços odontológicos (pretos: RP = 3,08; IC95% 1,06–8,95; pardos: RP = 3,78; IC95% 1,49–9,59) e avaliação negativa do atendimento (pretos: RP = 1,63; IC95% 1,08–2,45; pardos: RP = 1,64; IC95% 1,12–2,39). Discriminação racial se associou à falta de acesso (RP = 4,34; IC95% 1,45–12,95), ao não uso recente dos serviços (RP = 1,74; IC95% 1,31–2,31) e à avaliação negativa do atendimento (RP = 1,51; IC95% 1,01–2,25). E residir em vizinhanças com menos problemas físicos e sociais foi associado à menor prevalência de autopercepção negativa (RP = 0,70; IC95% 0,54–0,90), ao comprometimento da saúde bucal (RP = 0,87; IC95% 0,76–0,99), à falta de acesso (RP = 0,42; IC95% 0,18–0,98) e à avaliação negativa do atendimento (RP = 0,56; IC95% 0,40–0,79).

**CONCLUSÕES:**

Os achados evidenciaram que o racismo impacta a saúde bucal por vias estruturais e interpessoais e destacam a urgência de estratégias amplas e eficazes para reduzir as iniquidades que afetam os grupos racializados.

## INTRODUÇÃO

As iniquidades raciais em saúde bucal são amplamente documentadas, com evidências indicando que indivíduos de grupos racializados enfrentam maiores barreiras no acesso e na qualidade dos cuidados odontológicos, além de apresentarem maior prevalência de condições como cárie, doença periodontal e perda dentária^1,2^. A literatura tem destacado a importância de compreender a raça como um construto social e de investigar o racismo como causa fundamental dessas iniquidades^3,4^.

O racismo, enquanto sistema social complexo, opera em múltiplos níveis. No nível estrutural, expressa-se na organização histórica e institucional da sociedade, por meio de políticas, normas, práticas e arranjos institucionais que produzem e reproduzem hierarquias raciais, condicionando o acesso desigual a recursos, oportunidades e direitos. No nível interpessoal, manifesta-se em atitudes e comportamentos discriminatórios que afetam diretamente indivíduos racializados, perpetuando experiências de exclusão e subordinação^5^. Esse sistema impacta a saúde por diversas vias, incluindo: (I) a injustiça econômica e a segregação social, que geram condições precárias de vida; (II) a exposição desigual a ambientes físicos e sociais adversos; e (III) o trauma psicossocial resultante da discriminação interpessoal, com efeitos sobre a saúde mental, comportamental e física^6^. Assim, o local de residência e as experiências de vida estão intimamente ligados ao estado de saúde dos indivíduos^3^.

Apesar das evidências consistentes sobre as iniquidades raciais em saúde bucal, o papel do racismo na produção dessas iniquidades permanece subexplorado. A maioria dos estudos adota abordagens descritivas baseadas unicamente na raça autoidentificada, o que restringe a compreensão dos mecanismos pelos quais o racismo influencia os desfechos em saúde bucal^3^. Investigações que avancem além dessa perspectiva e examinem as vias de influência do racismo sobre a saúde bucal e o acesso aos serviços são essenciais para orientar políticas públicas voltadas à equidade.

Nesse contexto, o presente estudo teve como objetivo investigar o impacto do racismo na saúde bucal e no acesso a serviços odontológicos, considerando duas dimensões desse sistema: o racismo interpessoal, mensurado pela experiência de discriminação racial autorreferida, e o racismo estrutural, representado pelas características do ambiente de vizinhança, que expressam a segregação residencial, a desigualdade socioespacial e a concentração histórica de desvantagens. A compreensão dessas relações contribui para identificar mecanismos que produzem as iniquidades raciais em saúde bucal e fornecer subsídios para políticas públicas e estratégias de intervenção mais equitativas.

## MÉTODOS

### Desenho, População do Estudo e Aspectos Éticos

Foi realizado um estudo transversal com estudantes de graduação matriculados em 2023 na Universidade Federal de Juiz de Fora (UFJF), instituição pública com sede em Juiz de Fora (MG) e *campus* avançado em Governador Valadares (MG). Foram elegíveis todos os estudantes com 18 anos ou mais, sendo considerados perda amostral aqueles que não responderam ao questionário de pesquisa após três tentativas de contato.

A escolha da população universitária fundamenta-se pela diversidade racial, socioeconômica e territorial presente no ambiente acadêmico, o que favorece a análise de iniquidades raciais em um contexto institucional plural. Embora ainda marcado por desigualdades, o espaço universitário tornou-se mais heterogêneo com a implementação de ações afirmativas. Esse cenário permite investigar a persistência das iniquidades raciais mesmo em contextos de maior escolaridade, além de evidenciar os efeitos do racismo em trajetórias marcadas por diferentes exposições e oportunidades sociais.

Em 2023, a UFJF contava com 15.361 estudantes matriculados em cursos presenciais de graduação, sendo 87,6% no *campus* de Juiz de Fora. Desses, 59,4% se autodeclaravam brancos, 27,5% pardos, 10,7% pretos e 2,4% amarelos, indígenas ou de outra raça/cor. O tamanho amostral foi calculado para populações finitas, estimando-se um mínimo de 347 participantes, com base em uma prevalência esperada de discriminação racial de 9%^7^, intervalo de confiança de 95% e erro amostral de 5%.

O estudo foi aprovado pelo Comitê de Ética em Pesquisa com Seres Humanos da UFJF (parecer nº 6.041.263).

### Coleta de Dados

A coleta de dados foi realizada por meio de questionário *online*, elaborado na plataforma Google Formulários e enviado por e-mail institucional aos estudantes. O instrumento continha 54 questões, organizadas em quatro blocos: Bloco I - Dados demográficos e socioeconômicos; Bloco II – Saúde bucal; Bloco III – Escala de Percepção de Vizinhança^8^; Bloco IV – Escala de Discriminação Explícita (EDE) abreviada^9^.

A Escala de Percepção de Vizinhança, validada no Brasil, avalia a percepção do ambiente considerando desordens físicas e sociais, com pontuação de 0 a 32, sendo que valores mais altos indicam maiores problemas na vizinhança^8^. A EDE abreviada é um instrumento validado que mensura a experiência de discriminação explícita no contexto brasileiro. Sua versão abreviada, considerada adequada para estudantes de graduação^9^, mede experiências de discriminação em oito situações cotidianas, com escores variando de 0 a 24, sendo que valores mais altos indicam maior discriminação percebida.

### Variáveis

Na presente investigação, adotou-se uma estratégia analítica voltada a articular diferentes dimensões do racismo. A caracterização do ambiente de vizinhança foi utilizada como *proxy* do racismo estrutural, considerando que as desigualdades territoriais expressam a materialização histórica e institucional das hierarquias raciais no espaço urbano^6^. Já a discriminação autorreferida foi considerada uma medida do racismo interpessoal, refletindo as experiências diretas de tratamento diferenciado e de preconceito vivenciadas pelos indivíduos em interações cotidianas^9^. Essa operacionalização permitiu captar simultaneamente níveis distintos de manifestação do racismo (estrutural e interpessoal) e analisar seus efeitos sobre os desfechos em saúde bucal e no acesso a serviços odontológicos.

Dessa forma, as variáveis independentes incluíram: raça/cor, discriminação racial e caracterização da vizinhança.

A variável raça/cor foi obtida conforme a autodeclaração dos participantes, com base na classificação do Instituto Brasileiro de Geografia e Estatística (IBGE): branca, preta, parda, amarela e indígena. A discriminação racial foi mensurada por meio da versão abreviada da EDE, sendo dicotomizada em “sim” ou “não”. Considerou-se discriminado o indivíduo que relatou pelo menos um episódio de tratamento desigual motivado por raça/cor e o interpretou como discriminatório. A caracterização da vizinhança foi mensurada por meio da Escala de Percepção de Vizinhança e dicotomizada em “menos problemas físicos e sociais” (tercil inferior) e “mais problemas físicos e sociais” (tercis intermediário e superior)^10^. Considerando que muitos estudantes migram de cidade para cursar a graduação, considerou-se a vizinhança onde o participante residiu pela maior parte da vida ou, no mínimo, por cinco anos, a fim de capturar experiências de longo prazo que influenciaram suas oportunidades de vida.

Foram analisadas duas categorias de desfechos: desfechos relacionados à saúde bucal (autopercepção de saúde bucal, comprometimento da saúde bucal e perda dentária) e desfechos relacionados ao acesso e à percepção da qualidade de serviços odontológicos (acesso a serviços odontológicos, uso recente de serviços odontológicos e avaliação do atendimento).

A autopercepção da saúde bucal foi classificada em positiva ou negativa, conforme avaliação do próprio respondente. O comprometimento da saúde bucal foi identificado pela presença de pelo menos uma das seguintes condições: dor de dente, desconforto com a aparência bucal ou dificuldades alimentares, considerando-se as respostas “muito frequentemente”, “frequentemente” ou “às vezes” como indicativas de comprometimento^11^. E a perda dentária foi definida pelo relato de extração de pelo menos um dente permanente, sendo categorizada em “sim” e “não”.

As variáveis relacionadas ao acesso e à percepção da qualidade de serviços odontológicos foram baseadas no questionário do Projeto SB Brasil 2020. O acesso foi classificado em “acesso” para as respostas “procurei e fui agendado” ou “fui atendido”; e “falta de acesso” para a resposta “não fui atendido”. O uso recente de serviços odontológicos foi definido como a realização de consulta no último ano, enquanto o não uso recente incluiu os casos em que a última consulta ocorreu há mais de um ano ou quando o indivíduo nunca havia ido ao dentista. A avaliação do atendimento odontológico foi categorizada como “positiva” ou “negativa”.

As covariáveis incluíram idade, gênero, estado conjugal, renda familiar, escolaridade materna, atividade remunerada, tipo de escola no ensino médio, recebimento de auxílio estudantil e área de conhecimento do curso.

### Análises Estatísticas

As análises foram realizadas no *software* R (versão 4.2.1). Inicialmente, procedeu-se à análise descritiva. Para estimar as razões de prevalência, utilizou-se regressão de Poisson com variância robusta. Esta abordagem é recomendada em estudos transversais com desfechos binários, pois fornece estimativas mais precisas das razões de prevalência^12^. Adotou-se intervalos de confiança de 95% (IC95%).

As variáveis incluídas nos modelos ajustados foram selecionadas com base em diagramas acíclicos direcionados (DAGs), construídos no programa DAGitty, conforme pressupostos teóricos e evidências empíricas da literatura^4^. Os DAGs foram construídos a partir de uma adaptação do modelo proposto por Howe et al.^4^, que originalmente abordou desfechos de saúde distintos (comportamentos de saúde, índice de massa corporal – IMC e condições físicas). Para o presente estudo, foram realizadas adaptações conceituais e analíticas para o contexto da saúde bucal e do acesso a serviços odontológicos, permitindo representar relações causais específicas desses desfechos. Variáveis latentes foram incorporadas para capturar fatores não observáveis com possível influência causal. Variáveis contextuais relativas à oferta de serviços (que podem influenciar o acesso e a saúde bucal) não foram incluídas nos diagramas devido à indisponibilidade desses dados na população de estudo.

Os ajustes dos modelos de regressão de Poisson foram avaliados em relação à presença de sobredispersão (quando a variância é maior do que a média, identificada pela estatística de dispersão > 1,2), à presença de multicolinearidade (pela estatística VIF > 5, “*variance inflation factor*”) e pela distribuição dos resíduos de Pearson e Deviance sem grandes desvios (dentro da variação de -0,5 +0,5).

A Figura apresenta o DAG com exposições, desfechos e covariáveis do estudo.

**Figura:** Diagrama causal das relações entre exposições, desfechos e covariáveis do estudo.

## RESULTADOS

O estudo incluiu 686 estudantes de graduação. As categorias amarela e indígena foram excluídas das análises devido à baixa representatividade (um indígena e nenhum amarelo). A distribuição etária foi de 17,9% entre 18 e 20 anos, 51,9% entre 21 e 24 anos e 30,2% com 25 anos ou mais. A maioria era do sexo feminino (67,9%) e possuía renda familiar mensal de até três salários mínimos (47,7%). Quanto à raça/cor, 58,2% se autodeclararam brancos, 23,2% pardos e 18,6% pretos.

A Tabela 1 apresenta as características da população do estudo, estratificadas por raça/cor. Houve maior proporção de indivíduos pretos e pardos nas categorias de menor renda familiar, em comparação aos brancos (p < 0,001). A frequência de matrícula em escolas públicas no ensino médio também foi significativamente mais elevada entre pretos e pardos (p < 0,001). Pretos e pardos relataram maior frequência de residência em vizinhanças com mais problemas físicos e sociais, em comparação aos brancos (p < 0,001). E a prevalência de discriminação racial referida também foi maior entre indivíduos pretos (p < 0,001).

**Tabela 1:** Características da população de estudo: distribuição total e por raça/cor, em valores absolutos e percentuais (n = 686).

Variável	Total		Raça/cor						p valor
			Branca		Parda		Preta		
	n	%	n	%	n	%	n	%	
Gênero									0,283
Feminino cisgênero	466	67,9	261	56,0	116	24,9	89	19,1	
Masculino cisgênero	189	27,6	118	62,4	35	18,5	36	19,0	
Transgênero, agênero ou não binário	31	4,5	20	64,5	8	25,8	3	9,7	
Idade									0,097
Menos de 20 anos	123	17,9	72	58,5	32	26,0	19	15,4	
Entre 20 e 24 anos	356	51,9	221	62,1	71	19,9	64	18,0	
Acima de 25 anos	207	30,2	106	51,2	56	27,1	45	21,7	
Estado conjugal									0,073
Com cônjuge	76	11,1	36	47,4	25	32,9	15	19,7	
Sem cônjuge	610	88,9	363	59,5	134	22,0	113	18,5	
Renda familiar mensal									< 0,001
De 0 a 3 salários mínimos	327	47,7	142	43,4	88	26,9	97	29,7	
Entre 3 e 5 salários mínimos	158	23,0	108	68,4	35	22,2	15	9,5	
5 salários mínimos ou mais	201	29,3	149	74,1	36	17,9	16	8,0	
Escolaridade materna									< 0,001
Até ensino fundamental completo	169	24,6	75	44,4	55	32,5	39	23,1	
Ensino médio incompleto ou completo	223	32,5	120	53,8	49	22,0	54	24,2	
Ensino superior incompleto ou mais	294	42,9	204	69,4	55	18,7	35	11,9	
Atividade remunerada									0,246
Não	501	73,0	301	60,1	111	22,2	89	17,8	
Sim	185	27,0	98	53,0	48	25,9	39	21,1	
Tipo de escola no ensino médio									< 0,001
Privada	282	41,1	215	76,2	40	14,2	27	9,6	
Pública	404	58,9	184	45,5	119	29,5	101	25,0	
Ingresso na UFJF por cotas raciais									< 0,001
Não	532	77,6	399	75,0	89	16,7	44	8,3	
Sim	154	22,4	0	0	70	45,5	84	54,5	
Auxílio estudantil									< 0,001
Não	554	80,8	343	61,9	126	22,7	85	15,3	
Sim	132	19,2	56	42,4	33	25,0	43	32,6	
Área de conhecimento do curso									0,556
Ciências da Saúde e Biológicas	241	35,1	141	58,5	55	22,8	45	18,7	
Ciências Exatas e Engenharias	91	13,3	60	65,9	17	18,7	14	15,4	
Ciências Humanas e Sociais	354	51,6	198	55,9	87	24,6	69	19,5	
Experiência de discriminação racial									
Não	436	63,6	356	81,7	72	16,5	8	1,8	< 0,001
Sim	250	36,4	43	17,2	87	34,8	120	48,0	
Caracterização da vizinhança									< 0,001
Mais problemas	309	45,0	153	49,5	85	27,5	71	23,0	
Menos problemas	377	55,0	246	65,3	74	19,6	57	15,1	

UFJF: Universidade Federal de Juiz de Fora.

Entre os estudantes que relataram discriminação interpessoal, 92,3% atribuíram o tratamento desigual a múltiplos motivos, sendo os mais citados a forma de vestir, condição socioeconômica e raça/cor. Especificamente quanto à discriminação racial, 36,4% relataram essa experiência. As ocorrências mais frequentes incluíram: tratamento inferior em estabelecimentos comerciais (21,0%), ser confundido com funcionário (17,1%) e ofensas verbais (18,1%). Também foram referidas discriminações em instituições de ensino (12,1%), em relações afetivas (10,9%), na vizinhança (7,4%), em repartições públicas (6,7%) e no âmbito familiar (6,9%).

A Tabela 2 apresenta os desfechos em saúde bucal e acesso a serviços odontológicos. Houve comprometimento da saúde bucal em 57,3% dos participantes; 25,7% relataram autopercepção negativa e 16,5%, perda dentária. Quanto ao acesso, 3,9% indicaram falta de acesso; 69,0% haviam utilizado serviços odontológicos no último ano, e 17,3% avaliaram negativamente o atendimento recebido.

**Tabela 2:** Prevalências dos desfechos em saúde bucal e acesso a serviços odontológicos: distribuição total e por raça/cor (n = 686).

Desfecho	Total		Raça/cor					
			Branca		Parda		Preta	
	n	% (IC95%)	n	% (IC95%)	n	% (IC95%)	n	% (IC95%)
Saúde bucal								
Autopercepção negativa de saúde bucal	176	25,7 (22,4–29,0)	81	20,3 (16,4–24,2)	52	32,7 (25,4–40,0)	43	33,6 (25,4–41,8)
Comprometimento da saúde bucal	393	57,3 (53,6–61,0)	225	56,4 (51,5–61,3)	92	57,9 (50,3–65,5)	76	59,4 (51,0–67,8)
Perda dentária	113	16,5 (13,8–19,2)	50	12,5 (9,2–15,8)	30	18,9 (12,8–25,0)	33	25,8 (18,2–33,4)
Acesso a serviços odontológicos								
Falta de acesso a serviços odontológicos	27	3,9 (2,5–5,3)	7	1,8 (0,4–3,2)	12	7,5 (3,4–11,6)	8	6,3 (2,2–10,4)
Ausência de uso recente de serviços odontológicos	213	31,0 (27,5–34,5)	114	28,6 (24,1–33,1)	52	32,7 (25,4–40,0)	47	36,7 (28,3–45,1)
Avaliação negativa do atendimento odontológico	119	17,3 (14,6–20,0)	52	13,0 (9,7–16,3)	37	23,3 (16,6–30,0)	30	23,4 (16,1–30,7)

IC95%: intervalo de confiança de 95%.

A Tabela 3 apresenta as razões de prevalência (RP) entre as variáveis de exposição e os desfechos relacionados à saúde bucal. Após ajuste para confundidores indicados pelo DAG, a raça/cor manteve-se associada (p < 0,05) com autopercepção negativa da saúde bucal (RP = 1,56 IC95% 1,14–2,13 para pretos e RP 1,53 IC95% 1,14–2,05 para pardos) e perda dentária (RP = 1,99 IC95% 1,33–2,97 para pretos). A experiência de discriminação racial associou-se a piores desfechos de saúde bucal apenas na análise bruta, com associação com autopercepção negativa da saúde bucal (RP = 1,49; IC95% 1,16–1,91) e perda dentária (RP = 1,78; IC95% 1,27–2,48). Por fim, morar em áreas com menos problemas físicos e sociais foi fator protetor para autopercepção negativa (RP = 0,70; IC95% 0,54–0,90) e comprometimento da saúde bucal (RP = 0,87; IC95% 0,76–0,99), mesmo após ajuste para fatores confundidores (p < 0,05).

**Tabela 3:** Razões de prevalência dos desfechos relacionados à saúde bucal por raça/cor, experiência de discriminação racial e caracterização da vizinhança (n = 686).

Variável	Autopercepção negativa de saúde bucal		Comprometimento da saúde bucal		Perda dentária	
	RP bruta (IC95%)	RP ajustada (IC95%)	RP bruta (IC95%)	RP ajustada (IC95%)	RP bruta (IC95%)	RP ajustada (IC95%)
Raça/cor^a^						
Branca	1	1	1	1	1	1
Parda	1,61 (1,20–2,17)	1,53 (1,14–2,05)	1,03 (0,88–1,20)	1 (0,86–1,18)	1,51 (1,00–2,28)	1,46 (0,96–2,23)
Preta	1,65 (1,21–2,26)	1,56 (1,14–2,13)	1,05 (0,89–1,24)	1,03 (0,87–1,22)	2,06 (1,39–3,04)	1,99 (1,33–2,97)
Discriminação racial^b^						
Não	1	1	1	1	1	1
Sim	1,49 (1,16–1,91)	1,07 (0,77–1,47)	1,08 (0,94–1,23)	1,05 (0,87–1,25)	1,78 (1,27–2,48)	1,18 (0,78–1,79)
Caracterização da vizinhança^c^						
Mais problemas	1	1	1	1	1	1
Menos problemas	0,65 (0,50–0,84)	0,70 (0,54–0,90)	0,87 (0,76–0,99)	0,87 (0,76–0,99)	0,75 (0,54–1,05)	0,82 (0,58–1,16)

RP: razão de prevalência; IC95%: intervalo de confiança de 95%; DAG: diagramas acíclicos direcionados.

^a^ Razão de prevalência ajustada para raça pela caracterização da vizinhança, conforme DAG.

^b^ Razão de prevalência ajustada para discriminação racial por gênero, idade, caracterização da vizinhança e raça/cor, conforme DAG.

^c^ Razão de prevalência ajustada para caracterização da vizinhança pela raça/cor, conforme DAG.

Nota: os ajustes dos modelos de regressão de Poisson foram avaliados quanto à presença de sobredispersão (estatística de dispersão > 1,2), à multicolinearidade (VIF > 5) e pela distribuição dos resíduos de Pearson e Deviance, que se mantiveram próximos a zero, indicando bom ajuste do modelo.

Na Tabela 4, são apresentadas as associações com os desfechos de acesso e percepção de qualidade dos serviços odontológicos. Na análise ajustada, a raça/cor manteve associação (p < 0,05) com falta de acesso e avaliação negativa do atendimento. Indivíduos pretos (RP = 3,08; IC95% 1,06–8,95) e pardos (RP = 3,78; IC95% 1,49–9,59) apresentaram maior prevalência de falta de acesso e de avaliação negativa do atendimento recebido (pretos: RP = 1,63; IC95% 1,08–2,45; pardos: RP = 1,64; IC95% 1,12–2,39). A experiência de discriminação racial associou-se à falta de acesso (RP = 4,34; IC95% 1,45–12,95), a ausência de uso recente dos serviços (RP = 1,74; IC95% 1,31–2,31) e à avaliação negativa do atendimento (RP = 1,51; IC95% 1,01–2,25) (p < 0,05). E morar em vizinhanças com menos problemas foi fator protetor tanto para falta de acesso (RP = 0,42; IC95% 0,18–0,98), quanto para avaliação negativa do atendimento (RP = 0,56; IC95% 0,40–0,79) (p < 0,05). Ressalta-se que algumas das associações observadas, especialmente entre falta de acesso a serviços odontológicos, raça/cor e discriminação racial, embora tenham apresentado significância estatística, exibiram intervalos de confiança amplos, indicando menor precisão das estimativas. Essa imprecisão decorre, principalmente, da baixa prevalência do desfecho na população estudada, o que reduz o número de eventos e amplia a incerteza das medidas de efeito. Ainda assim, a consistência da direção das associações reforça a presença de iniquidades raciais no acesso aos serviços, mesmo diante de limitações amostrais e estatísticas.

**Tabela 4:** Razões de prevalência dos desfechos relacionados ao acesso a serviços odontológicos por raça/cor, experiência de discriminação racial e caracterização da vizinhança (n = 686).

Variável	Falta de acesso a serviços odontológicos		Ausência de uso recente de serviços odontológicos		Avaliação negativa do atendimento odontológico	
	RP bruta (IC95%)	RP ajustada (IC95%)	RP bruta (IC95%)	RP ajustada (IC95%)	RP bruta (IC95%)	RP ajustada (IC95%)
Raça/cor^a^						
Branca	1	1	1	1	1	1
Parda	4,30 (1,72–10,7)	3,78 (1,49–9,59)	1,14 (0,87–1,50)	1,14 (0,87–1,50)	1,79 (1,22–2,61)	1,64 (1,12–2,39)
Preta	3,56 (1,32–9,63)	3,08 (1,06–8,95)	1,29 (0,98–1,69)	1,28 (0,97–1,69)	1,80 (1,20–2,69)	1,63 (1,08–2,45)
Discriminação racial^b^						
Não	1	1	1	1	1	1
Sim	6,1 (2,50–14,92)	4,34 (1,45–12,95)	1,54 (1,24–1,92)	1,74 (1,31–2,31)	1,96 (1,42–2,71)	1,51 (1,01–2,25)
Caracterização da vizinhança^c^						
Mais problemas	1	1	1	1	1	1
Menos problemas	0,35 (0,15–0,78)	0,42 (0,18–0,98)	0,96 (0,77–1,2)	0,99 (0,79–1,24)	0,52 (0,37–0,72)	0,56 (0,40–0,79)

RP: razão de prevalência; IC95%: intervalo de confiança de 95%; DAG: diagramas acíclicos direcionados.

^a^ Razão de prevalência ajustada para raça pela caracterização da vizinhança, conforme DAG.

^b^ Razão de prevalência ajustada para discriminação racial por gênero, idade, caracterização da vizinhança e raça/cor, conforme DAG.

^c^ Razão de prevalência ajustada para caracterização da vizinhança pela raça/cor, conforme DAG.

Nota: os ajustes dos modelos de regressão de Poisson foram avaliados quanto à presença de sobredispersão (estatística de dispersão > 1,2), à multicolinearidade (VIF > 5) e pela distribuição dos resíduos de Pearson e Deviance, que se mantiveram próximos a zero, indicando bom ajuste do modelo.

## DISCUSSÃO

Os achados deste estudo confirmam iniquidades raciais significativas na saúde bucal e no acesso a serviços odontológicos, com o racismo operando de forma interpessoal e estrutural na sua produção. Observou-se que indivíduos que se autodeclararam pretos apresentaram maior prevalência de autopercepção negativa da saúde bucal, perda dentária, falta de acesso aos serviços odontológicos e avaliação negativa do atendimento, em comparação aos brancos. Entre os indivíduos pardos, verificou-se maior prevalência de autopercepção negativa da saúde bucal, falta de acesso aos serviços odontológicos e avaliação negativa do atendimento, também em relação aos brancos.

A filiação a grupos raciais historicamente marginalizados expõe os indivíduos a maiores níveis de discriminação interpessoal e a contextos sociais adversos, ambos com impacto na saúde^4^. Neste estudo, a discriminação racial associou-se aos desfechos bucais apenas na análise bruta, perdendo significância após ajuste, um resultado que dialoga com a heterogeneidade observada na literatura. Enquanto estudos como os de Celeste et al.^13^ e Finlayson et al.^14^ não observaram associações significativas, outros estudos, como os de McGlumphy et al.^15^, Cozier et al.^16^ e Raskin et al.^17^, identificaram relação positiva entre experiências de discriminação racial e piores desfechos em saúde bucal, como autopercepção negativa e perda dentária. Tais efeitos podem ser mediados pelo sofrimento psicológico e pela adoção de comportamentos nocivos à saúde, como tabagismo, consumo abusivo de álcool e ingestão excessiva de açúcares, além de restrições ao acesso a oportunidades educacionais, laborais e habitacionais, que ampliam a exposição a riscos ambientais e limitam o uso de serviços odontológicos^18^.

Este estudo também contribui para o entendimento dos efeitos da discriminação racial no acesso aos serviços odontológicos. Verificou-se associação entre experiências de discriminação, menor uso dos serviços e avaliações negativas do atendimento, em consonância com estudos prévios^17,19^. A discriminação pode afetar a percepção dos usuários sobre o sistema de saúde, prejudicar as interações com os profissionais e comprometer tanto o acesso quanto a qualidade dos cuidados. Situações discriminatórias em contextos clínicos tendem a gerar sentimentos de alienação, reforçar práticas paternalistas ou coercitivas e impor barreiras culturais e comunicacionais, o que alimenta a desconfiança no sistema, reduz a adesão ao tratamento e contribui para a insatisfação e a persistência de necessidades não atendidas^19^.

É importante destacar que tais atitudes não ocorrem isoladamente, mas refletem estruturas sociais que sustentam o racismo. Para além de comportamentos individuais, o racismo está enraizado na organização política e econômica da sociedade, operando como norma social reproduzida por estruturas de poder historicamente estabelecidas^3^. A literatura reconhece o racismo estrutural como o determinante central das iniquidades raciais em saúde^6^. Nesse sentido, compreender tais iniquidades exige atenção às condições históricas e contextuais que as sustentam, entre as quais a segregação residencial se destaca como um mecanismo fundamental do racismo estrutural^20^.

No Brasil, embora a segregação residencial não seja instituída por políticas formais de segregação racial, ela se manifesta amplamente por meio da exclusão de populações negras das áreas centrais e mais desenvolvidas das cidades^20^. Assim, as populações racializadas, frequentemente em situação de vulnerabilidade socioeconômica, tendem a residir em territórios marcados pela escassez de recursos, como alimentação saudável, educação de qualidade e oportunidades de emprego, e enfrentam maiores obstáculos no acesso a serviços de saúde adequados^21^.

Dessa forma, o ambiente de vizinhança configura-se como um marcador relevante para compreender os efeitos do racismo estrutural na saúde. Indivíduos que residem em um mesmo bairro compartilham condições físicas, sociais e institucionais que afetam sua saúde, para além de suas características individuais^22^. No estudo do racismo estrutural, a concentração espacial de comportamentos e desfechos de saúde em áreas hiperlocais reflete o contexto do bairro, fortemente influenciado pela segregação racial e pelas práticas institucionais que historicamente organizaram o espaço urbano segundo linhas raciais^23^. Mohottige et al.^24^ demonstraram que indicadores de racismo estrutural a nível de bairro, como segregação racial e privação socioeconômica, estavam associados a maior prevalência de doenças crônicas, incluindo diabetes, hipertensão e doença renal crônica, reforçando o papel da vizinhança como expressão concreta do racismo estrutural.

Os achados do presente estudo confirmam que viver em áreas com melhores condições físicas e sociais funciona como fator de proteção para desfechos negativos de saúde bucal, como autopercepção negativa e comprometimento da saúde bucal, bem como para barreiras de acesso a serviços odontológicos, incluindo falta de acesso e avaliação negativa do atendimento, mesmo após ajuste para raça/cor. A literatura sustenta que a residência em áreas desfavorecidas está frequentemente associada a um desequilíbrio entre fatores de proteção e de risco para a saúde. Esses territórios tendem a apresentar menor disponibilidade de serviços odontológicos, infraestrutura precária e uma sobrecarga de fatores de risco para doenças bucais, criando um cenário de vulnerabilidade que compromete a qualidade de vida de seus moradores^25,26^. A escassez de profissionais e unidades de atendimento, somada a barreiras financeiras, institucionais e culturais, dificulta o acesso a cuidados preventivos e curativos de qualidade. Ademais, a ausência de políticas públicas eficazes voltadas à distribuição equitativa dos serviços de saúde agrava essas iniquidades, reforçando iniquidades raciais e socioeconômicas no acesso à atenção odontológica^25,27^. Esses impactos são intensificados pela exposição a fatores de risco ambientais e comportamentais, como dietas ricas em açúcares, limitação da educação em saúde e prevalência de hábitos nocivos, como o tabagismo e o consumo excessivo de álcool, que também contribuem para o aumento do risco de doenças bucais^26^.

Portanto, a vizinhança deve ser compreendida não apenas como um contexto de vida, mas como expressão concreta das desigualdades estruturais reproduzidas historicamente, que moldam oportunidades, recursos e condições de saúde^22^. Ao refletir a segregação racial e a organização desigual do espaço urbano, o ambiente de vizinhança atua como um mediador fundamental das iniquidades em saúde bucal e no acesso a serviços odontológicos entre diferentes grupos raciais.

Ademais, é preciso também reconhecer que o racismo não atua isoladamente, mas se entrelaça com outras formas de opressão para moldar a saúde bucal e o acesso a serviços odontológicos^3^. A perspectiva interseccional permite compreender essas interações complexas, evidenciando como múltiplos eixos de opressão se combinam para impactar grupos historicamente marginalizados^11^. Estudos enfatizam que o racismo, o sexismo, o classismo e outros sistemas de marginalização estão interligados, co-constituindo uns aos outros e sendo centrais para as iniquidades raciais e não raciais em saúde^11,28^. Assim, indivíduos marginalizados em múltiplos eixos de opressão frequentemente dispõem de menos poder, menor controle sobre seus ambientes, capacidade reduzida de evitar contextos opressivos e menos recursos para lidar com a opressão, fatores que, em conjunto, podem explicar os efeitos negativos na saúde observados em alguns grupos^11^. Essa compreensão reforça a urgência de políticas públicas e intervenções que compreendam e enfrentem as múltiplas formas de opressão, promovendo equidade em saúde bucal.

Algumas limitações deste estudo devem ser consideradas. Por se basear em desfechos autorreferidos, há risco de viés de resposta e de memória, o que pode comprometer a acurácia das informações relatadas. A utilização de uma amostra não probabilística limita a generalização dos achados para a população universitária como um todo. E o delineamento transversal impede a inferência de relações causais diretas. Apesar disso, o estudo oferece contribuições relevantes ao evidenciar os efeitos do racismo interpessoal e estrutural sobre a saúde bucal e o acesso aos serviços odontológicos. Ao considerar duas importantes vias de impacto do racismo, o estudo amplia a compreensão dos determinantes que sustentam as iniquidades raciais nesse campo.

Os achados evidenciaram que o racismo estrutural e interpessoal dificulta o acesso a cuidados odontológicos de qualidade para os grupos raciais marginalizados e contribuem para a piora da condição de saúde bucal. A superação dessas iniquidades exige uma abordagem abrangente, voltada à transformação das condições sociais e econômicas que as perpetuam. Isso inclui a formulação e implementação de políticas públicas que combatam práticas discriminatórias, enfrentem a segregação espacial, promovam ambientes de vizinhança saudáveis e garantam a oferta equitativa de serviços odontológicos de qualidade, com atenção especial às populações historicamente excluídas. Somente por meio de mudanças estruturais será possível mitigar as iniquidades raciais em saúde bucal e avançar em direção a uma sociedade mais justa e equitativa.
